# Observation of five types of leaders contained in a negative triggered lightning

**DOI:** 10.1038/s41598-022-10366-x

**Published:** 2022-04-15

**Authors:** Jianguo Wang, Rui Su, Junlin Wang, Fukun Wang, Li Cai, Yating Zhao, Yijun Huang

**Affiliations:** School of Electrical Engineering and Automation, Wuhan University, Wuhan, China

**Keywords:** Electrical and electronic engineering, Atmospheric dynamics, Natural hazards

## Abstract

A negative triggered lightning involving five types of leaders was recorded by high-speed camera using frame rate of 20,000 fps and fast antennas at different distances. Five types of leaders contained one upward positive stepped leader, one upward positive dart leader, ten downward negative dart leader, one bidirectional leader and three downward negative dart-stepped leaders were propagated successively in the same channel. The upward positive dart leader occurred after initial continuous current pulse with average 2-D speed of 1.40 × 10^6^ m/s and started second continuous current process. The bidirectional leader was transformed from decaying unidirectional leader and showed the unique electric field changes. Faster return strokes are found to be induced by downward leaders propagating evenly and deposit more positive charge in the following residual channel. The positive charge can inhibit the potential initiation of an upward positive leader and boost the propagation of the next downward negative leader.

## Introduction

Lightning leaders can be divided into two main classes: stepped leaders are leaders creating the discharge channel in virgin air, while dart leaders are leaders propagating along a decaying residual discharge channel created by the corresponding stepped leaders^1–4^. Stepped leader gets its name from the faint individual steps during its propagation. In negative cloud-to-ground (CG) lightning flashes, a stepped leader may propagate downwards with abundant branches from the cloud to initiate the first return stroke (RS) in downward lightning flashes, or propagate upwards with few branches from the top of a high structure or a triggering wire to lead an initial continuous current (ICC) process in upward lightning flashes^5–7^. The step length may range from 3 to 200 m (typically 50 m) with step interval varying from 5 to 100 μs^8–10^. The electric field of a step leader will present pulses prior to the RS, and these electric field pulses is provided to be caused by the luminous step formation^4,11^. Previous studies have reported speeds of stepped leaders ranging from 0.8 × 10^5^ to 19 × 10^5^ m/s^1,10,12^.

Dart leaders propagate in a continuous way with few branches instead of a stepwise way, responsible for its terminology. The absence of the step formation during the propagation of dart leaders makes the electric field waveform of dart leaders smoother than that of stepped leaders. Optical measurements have provided dart leader mean speeds varying from 5.5 × 10^5^ to 19 × 10^6^ m/s for natural lightning flashes and 1.1 × 10^7^ to 2.0 × 10^7^ m/s for triggered lightning flashes^13,14^.

In negative CG lightning flashes, most of the reported dart leaders propagated downwards from the cloud to initiate the subsequent RS in both downward and upward lightning flashes. The exception that a dart leader can propagate upwards from the ground, in negative CG lightning flashes, is observed by Li et al.^15^. In that case, the upward positive dart leader occurred after a RS in the residual channel and propagated continuously to lead a continuing current (CC) process. The 3-D speeds of the upward dart leader range from 3.72 × 10^6^ to 14.48 × 10^6^ m/s with the average value of 6.5 × 10^6^ m/s.

Schonland et al.^1^ pointed that slower dart leaders tend to be preceded by longer interstroke intervals. The longer interstroke intervals may also lead to the occurrence of dart-stepped leaders. Dart-stepped leaders are lightning leaders propagating in a stepwise way like a stepped leader but propagating in the residual channel instead of virgin air. This definition of the dart-stepped leader is in contrast to the use of the term to describe a dart leader that departs from the residual channel to become a stepped leader.

Bidirectional leaders developing in the residual channel, as a special type of dart leaders, can also initiate subsequent RSs. This type of bidirectional leaders starts almost immediately below of a decaying dart or dart-stepped leader which terminated before reaching the ground. These bidirectional leaders have been observed in both triggered lightning flashes and upward lightning flashes initiated from a high structure^16,17^.

As mentioned above, there are six types of lightning leaders that can initiate a RS or a CC process (including ICC) in negative CG lightning flashes. They are downward negative stepped leaders, upward positive stepped leaders, downward negative dart leaders, upward positive dart leaders, downward negative dart-stepped leaders and bidirectional leaders. In this paper, a negative triggered lightning flash that contains five different types of sixteen leaders is reported and analyzed based on the high-speed video and electric field changes. The relationships between leader speeds, time and channel structure are also analyzed. To our knowledge, this is the first report of a lightning flash containing up to five types of leaders. The second continuous current initiated by an upward dart leader and the bidirectional leader transformed from a decaying dart leader are also first observed here.

## Result

### Five types of leaders

**Figure 1:**
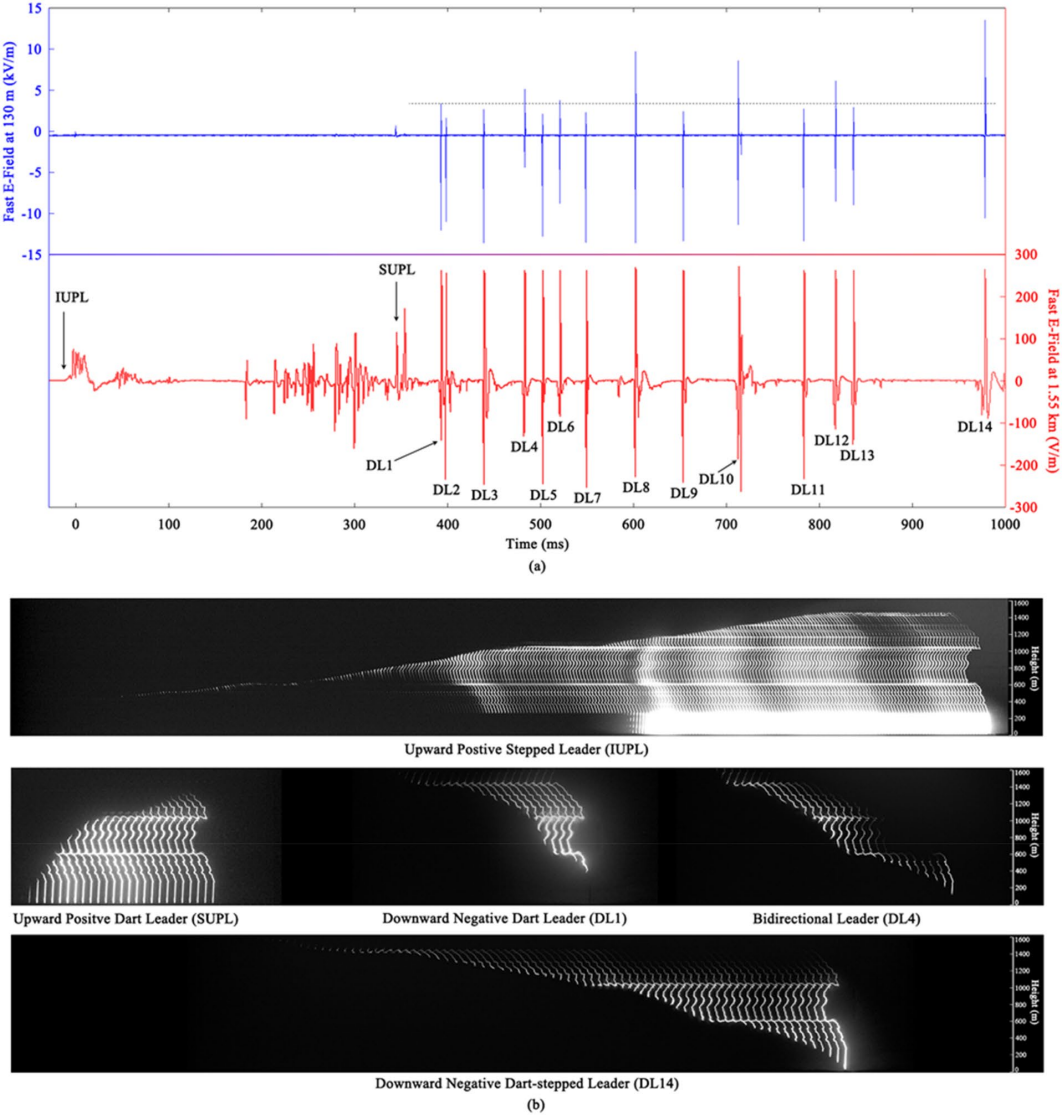
(**a**) The fast electric field changes of the F1907021515 at 130 m and 1.55 km. The electric field measurement antenna at 1.55 km is more sensitive and some parts of data are saturated. (**b**) The arranged high-speed frames showing the five types of lightning leader, with background removed and contrast enhanced. The arrange interval of IUPL is smaller than those of the other four leaders.

Figure 1a shows the fast electric field changes of the F1907021515 at 130 m and 1.55 km, and Fig. 1b shows the five types of leaders occurring during the flash. The time origin here is the trigger time of the electric field measurement antenna at 1.55 km. There are sixteen leaders initiating a CC or a RS process in this flash, whose characteristics are summarized in Table 1. The speeds in Table 1 are calculated between two successive frames based on the position of the leader head tip.

**Table 1:** Characteristics of the sixteen leaders in F1907021515.

ID	Occurrence time(ms)	Interstroke Interval (ms)	Speed (× 10^5^ m/s)			Speed variation	Type^b^
			Maximum	Minimum	Average		
IUPL	− 9.00	N/A	2.34	0.70	1.24	N/A	UPSL
SUPL	344.45	30^a^	27.22	5.14	13.98	N/A	UPDL
DL1	392.15	32	38.02	3.83	16.28	Fluctuation	DNDL
DL2	398.40	3	108.11	58.83	73.01	Acceleration	DNDL
DL3	438.90	31	96.54	20.61	46.57	Acceleration	DNDL
DL4	482.00	28	26.27^c^	6.80	16.80	Fluctuation	BL
DL5	502.05	13	85.55	22.60	51.46	Acceleration	DNDL
DL6	519.75	13	41.76	1.96	16.82	Fluctuation	DNDL
DL7	548.80	23	104.13	31.26	67.88	Acceleration	DNDL
DL8	601.75	44	35.25	13.83	24.51	Fluctuation	DNDL
DL9	653.45	36	102.09	20.31	57.13	Acceleration	DNDL
DL10	712.10	50	28.81	8.01	20.33	Fluctuation	DNDSL
DL11	783.40	54	67.38	28.94	50.34	Acceleration	DNDL
DL12	816.50	25	34.18	10.10	19.74	Fluctuation	DNDSL
DL13	836.55	13	53.35	10.91	30.86	Acceleration	DNDL
DL14	975.10	127	16.27	0.54	6.30	Fluctuation	DNDSL

^a^Interstroke Interval of SUPL is the interval between the occurrence of SUPL and the end of the last ICCP, instead of a RS.

^b^UPSL: upward positive stepped leader; UPDL: upward positive dart leader; DNDL: downward negative dart leader; BL: bidirectional leader; DNDSL: downward negative dart-stepped leader.

^c^The speed of DL4 represents the speed of its downward part.

The initial upward positive leader (IUPL) is a stepped leader initiating the ICC with fluent initial continuous current pulses (ICCPs) as shown by the electric field changes recorded at 1.55 km. This IUPL is a typical initial stepped leader in triggered lightning. IUPL has a faint body initially with the luminosity concentrated on its head. IUPL first occurs with noticeable luminosity at the height of about 420 m, obviously higher than the top of exploded triggering wire. The average step length of IUPL is 6.2 m, and the propagation of IUPL stagnates for several times. The plasma channel created by the exploded triggering wire becomes luminous when the head of IUPL reaches the height of about 1100 m.

Different from typical triggered lightning flashes that have one upward leader/continuing current process, F1907021515 has a second upward leader/continuing current process developing in the decaying channel of ICC. The upward leader here is a positive dart leader propagating in the residual channel in a continuous way with higher speeds. The upward dart leader initiates a CC process with one continuing current pulse (CCP). This second upward leader/continuing current process occurs before the dart leader/subsequent return stroke sequence, and hence we suggest this process belongs to the initial stage of this flash. Corresponding to the IUPL/ICC process, the upward dart leader is named second upward positive leader (SUPL), and the continuing current is named second continuous current (SCC).

The dart leader/subsequent return stroke sequence consists of 14 downward dart or dart-stepped leader (named DL1 to DL14) and corresponding return strokes. The sequence begins about 32 ms after the end of initial stage. Among these 14 downward leaders, DL4 is a bidirectional leader transformed from a dart leader propagate unidirectionally, DL10, DL12 and DL14 are three negative dart-stepped leaders, and the remained ten downward leaders are negative dart leaders. It can be found in Table 1 that the dart-stepped leaders DL10 and DL14 occur after relatively longer interstroke intervals of about 50 ms and 127 ms, respectively, while the dart-stepped leader DL12 occurs after a shorter interstroke intervals of 25 ms. DL1 to DL13 all propagate at speeds over 1 × 10^5^ m/s, while DL14 occurring after the longest interstroke interval once propagate at speeds on the order of 10^4^ m/s. The maximum speed values of the three dart-stepped leaders are less than those of ten dart leaders, while the average speed values not.

### Upward positive dart leader

**Figure 2:**
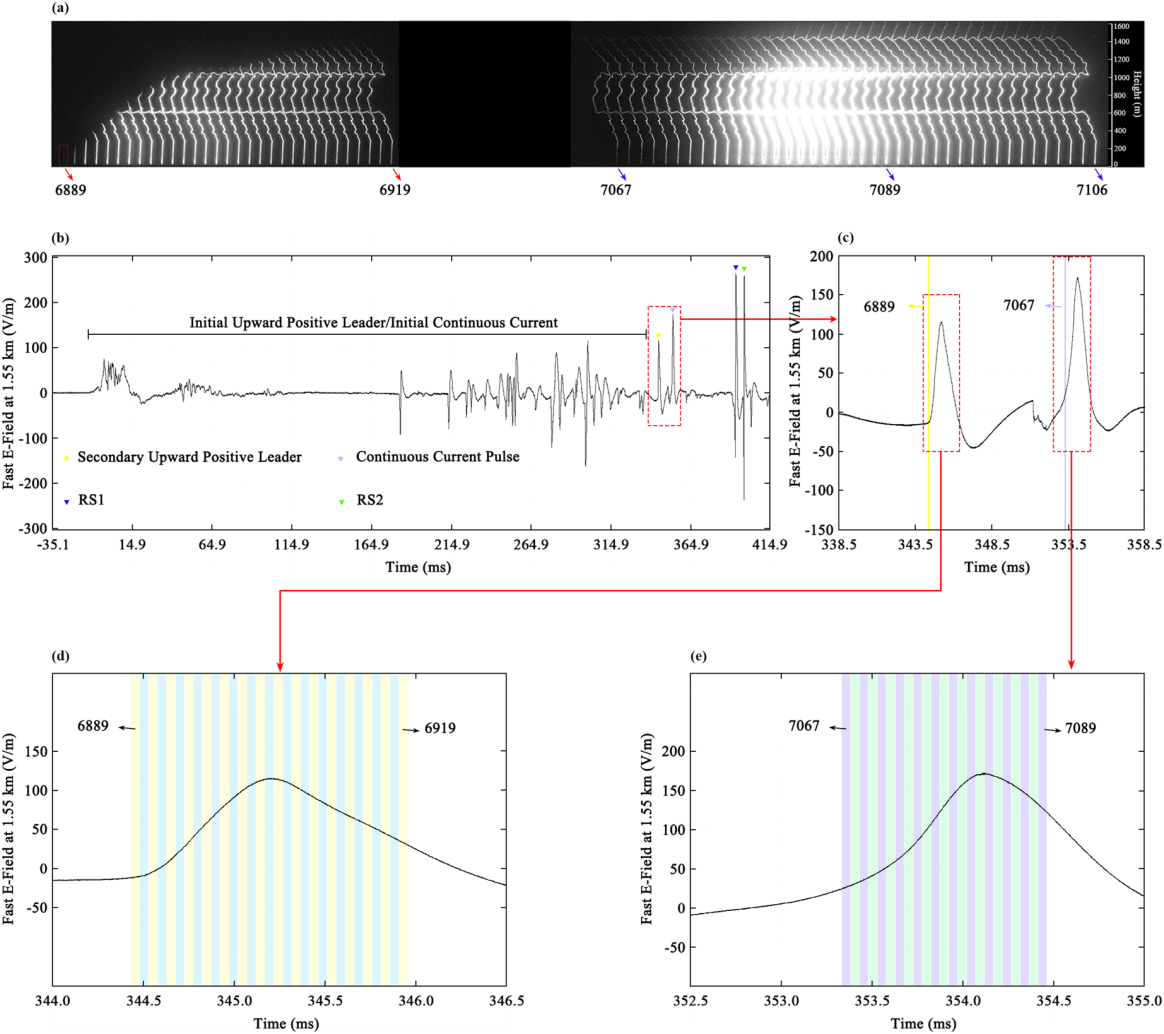
(**a**) Arranged high-speed frames showing the development of SUPL and SCCP. (**b**–**e**) The electric figure changes of SUPL and SCCP at 1.55 km expanded in different time scale. The colored triangles indicate the position of SUPL, SCCP, RS1 and RS2. The high-speed frames are represented by these yellow-and-cyan and purple-and-green rectangles respectively to match with the corresponding electric field changes with an uncertainty of 3.8 μs.

Figure 2 illustrates the development of SUPL and the following SCC with a continuing current pulse (SCCP). SUPL occurs in Frame 6889 or 344.45 ms (about 30 ms after the last ICCP), and illuminates the bottom of the residual channel. Before the occurrence of SUPL, there was no other obvious discharge process but the residual channel with slowly decaying luminosity recorded within FOV. SUPL is similar with the upward dart leader reported by Li et al.^15^ in that both of these two leaders initiate without any noticeable inducing factors. SUPL here propagates upward continuously with an average 2-D speed of 1.40 × 10^6^ m/s, which matches the speed range of dart leaders observed in natural lightning flashes. SCCP occurs from the Frame 7067 with a significant luminosity enhancement of the discharge channel.

The electric field changes at 1.55 km is expanded in different time scales in Fig. 2b–e. In Fig. 2b, the positive pulse peak of the SUPL is higher than that of the IUPL, and the positive pulse peak of SCCP is higher than those of all ICCPs but lower than those of RS1 and RS2. The electric field changes of SCC are expanded in a larger time scale in Fig. 2c. The durations corresponding to Frames 6889 and 7067 are indicated by a yellow rectangle and a purple rectangle, respectively. It can be seen that the initiation of SUPL is preceded by a slowly decreasing electric field change lasting over 6 ms with a peak of -16 V/m. The positive pulse peaks of SUPL and SCCP are about 116 V/m and 172 V/m, respectively. The electric field changes of SUPL and SCCP are further expanded in Fig. 2d and e, respectively. Frames 6889–6919 and Frames 7067–7089 are represented by these yellow-and-cyan and purple-and-green rectangles respectively to match with the corresponding electric field changes with an uncertainty of 3.8 μs. The occurrence of SUPL in Frame 6889 is near the watershed between the slow and the fast increase of the electric field. The positive pulse of SUPL reaches its peak in Frame 6904 when SUPL is still propagating upwards within FOV. This means the electric field has turned to decrease before SUPL reaches the cloud base. The electric field variation of SCCP coincides with the luminosity variation of the recorded channel. Both of the electric field and the luminosity of the channel are near the peak value from Frame 7080 to Frame 7087.

### Bidirectional leader

**Figure 3:**
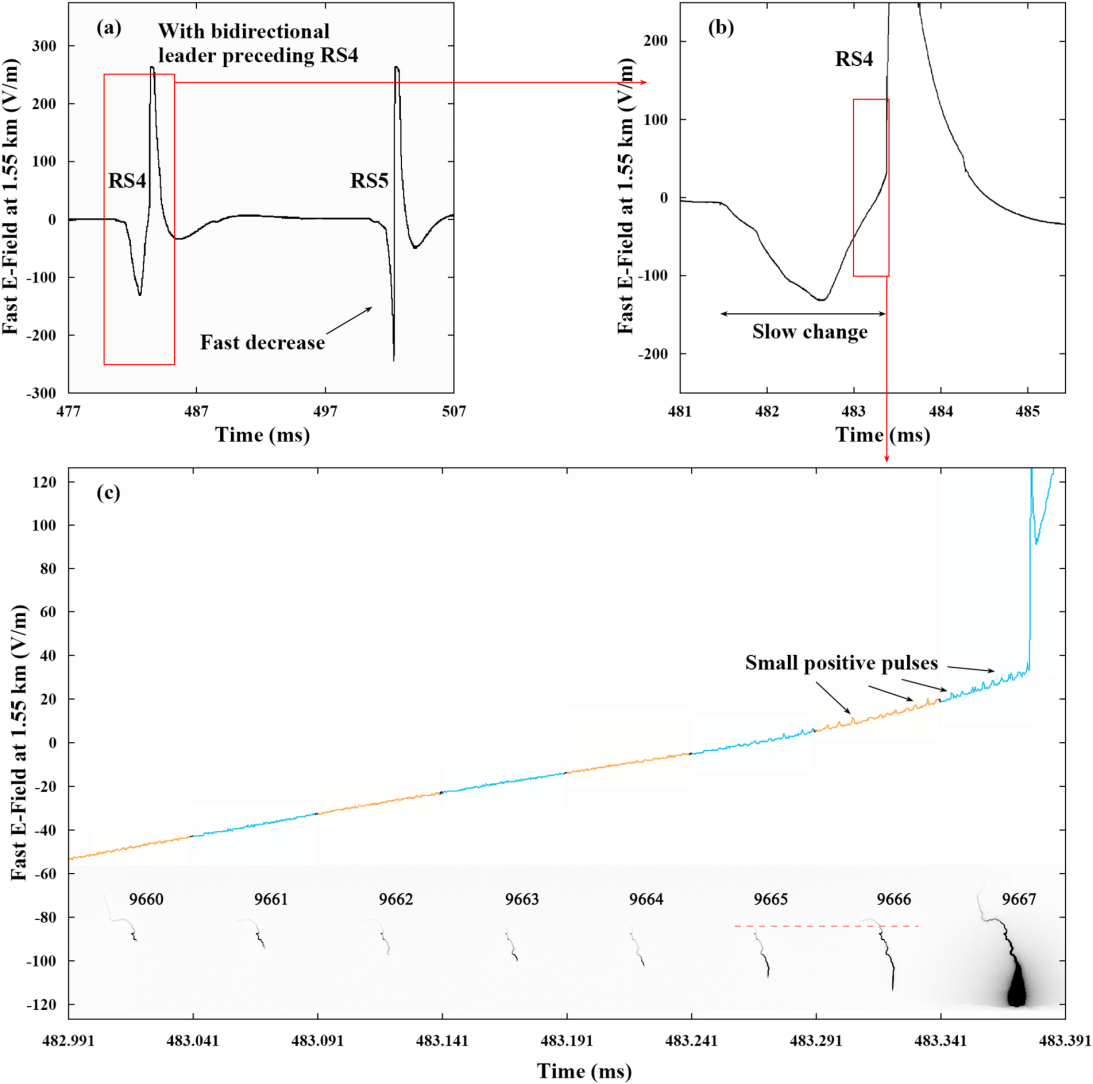
The electric field changes of DL4 at 1.55 km expanded in different time scale. The electric field waveform is colored to match corresponding high-speed frames with an uncertainty of 3.8 μs. The high-speed frames are background removed, intensity inverted and contrast enhanced to show the propagation process better.

Figure 3 illustrates the electric field changes of bidirectional leader (BL) DL4 before RS4 at 1.55 km expanded in different time scales with corresponding high-speed frames. In Fig. 3c, the electric field waveform is colored to match corresponding high-speed frames. DL4 occurs in FOV in Frame 9640 or about 28 ms after the RS3. DL4 has a “normal” initial propagation during which the luminous channel of DL4 extends downwards and gets longer. However, the luminous channel starts to get shorter with a fading luminosity from Frame 9655, which could be seen in the part of the DL4 in Fig. 1(b). The situation gets twist when the head of DL4 begins to extend in the channel of exploded triggering wire in Frame 9665. The channel near the tail of DL4 becomes more luminous in Frame 9665 and the position of the tail is clearly higher in Frame 9666, which indicate DL4 turns to propagate bidirectionally in Frame 9665.

The electric field changes showing the “slow change” preceding RS4 are different from those of the other rerun strokes showing the so called “fast decrease”^16^. The slow change preceding RS4 lasts about 2 ms with a negative peak value of about 133 V/m. The fast decrease preceding RS5 lasts less than 1 ms with the negative peak saturated. In Fig. 3c, with the waveform further expanded, it can be found that several small positive pulses superimposed on the slowly increasing waveform occur when DL4 begins to propagate bidirectionally.

These small positive pulses here are unipolar pulses whose peak values range from 1.01 to 4.36 V/m with the mean value of 2.34 V/m. The durations of these small positive pulses vary from 1 to 2 μs with the mean value of 1.24 μs. These small positive pulses are nonexistent before DL4 propagates bidirectionally or during the fast decrease processes preceding the other RSs. In addition, these small positive pulses are different from those pulses superimposed on the waveform of dart-stepped leaders, which will be discussed later. The slow change and small positive pulses superimposed on it are the common characteristics between DL4 and those two BLs observed in triggered lightning flashes by Qie et al.^16^.

**Figure 4:**
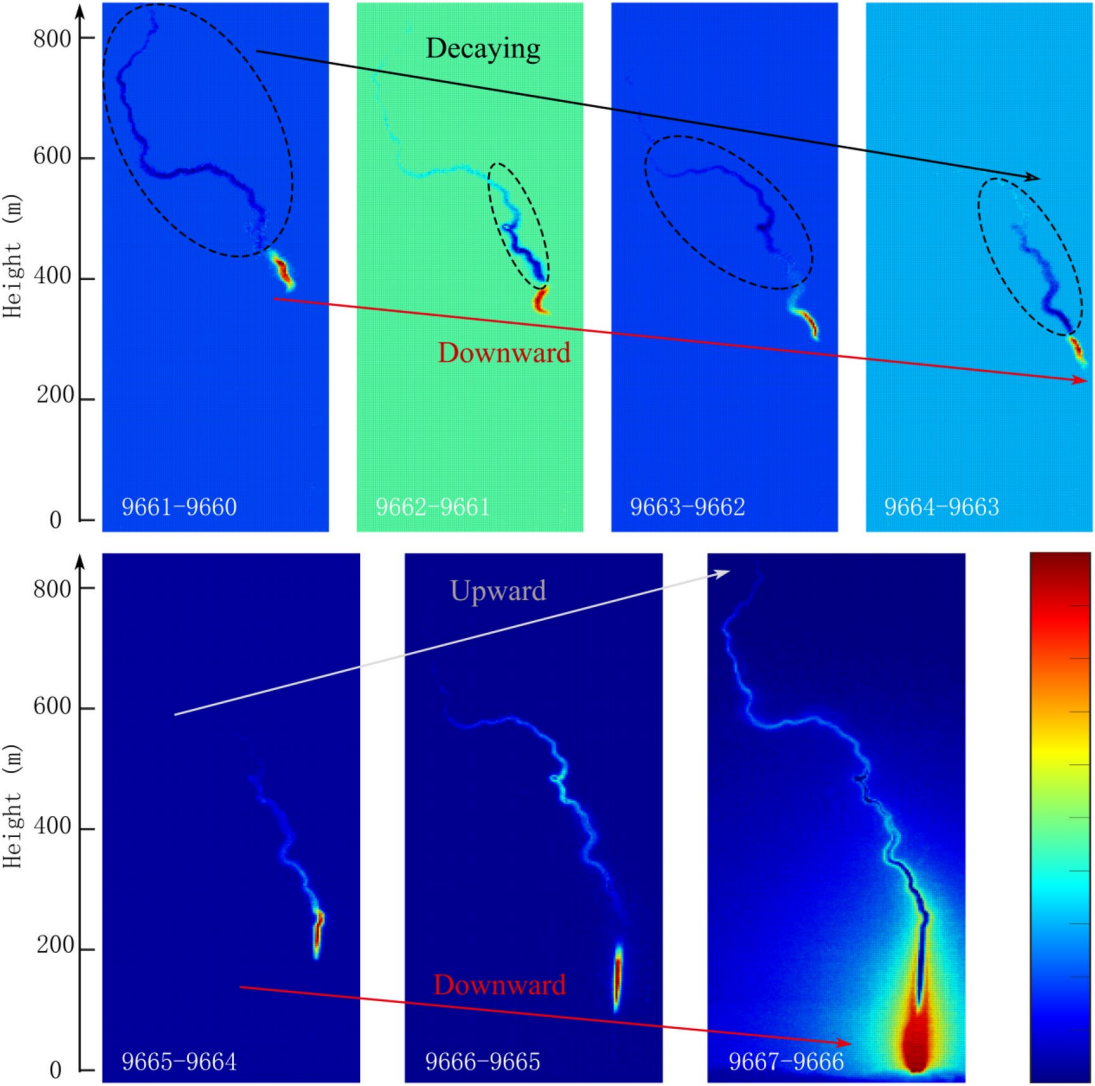
Differential luminosity from frame-to-frame of the frames in Fig. 3. The color range of each image is from the minimum value to the maximum value of the differential luminosity. The background color usually represents a differential light intensity of 0 which means that the luminosity of the corresponding position of the two frames remains unchanged. According to the color bar, redder means brighter and bluer means darker relative to the background color.

To achieve a better view and more clearly of the change process of DL4, Fig. 4 shows the differential luminosity from frame-to-frame of the frames in Fig. 3. Although the lower part had been developing downward, the upper part continued to get darken before Frame 9664. The DL4 with the decaying leader was significantly different from the sustained downward leader like DL1 Fig. 4 only showed the changes in the height of 800 m in a few frames near the beginning of the bidirectional leader. Actually, the upper channel under around 1500 m darkened from around Frame 9655. And the bright channel which had been significantly separated from the clouds base had getting shorter and shorter since then, indicating the discontinuous current. Until the leader began to develop bidirectionally, the decaying process lasted about 500 μs from Frame 9655 to Frame 9665. Such time interval is sufficient for the generation of bidirectional leader^16^.

### Dart stepped leader

There are three downward dart-stepped leaders (DL10, DL12 and DL14) featuring the relatively lower propagation speeds and abundant bipolar pulses superimposed on the electric field waveform during their propagation processes. The average propagation speeds of DL10, DL12 and DL14 are 20.33 × 10^5^, 19.74 × 10^5^ and 6.30 × 10^5^ m/s, respectively. DL4 has an average downward propagation speed of 16.80 × 10^5^ m/s, which is lower than those of two dart-stepped leaders (DL10 and DL12) here. However, there are few bipolar pulses superimposed on the electric field waveform of DL4 but some small unipolar positive pulses occurring during the bidirectional propagation process.

**Figure 5:**
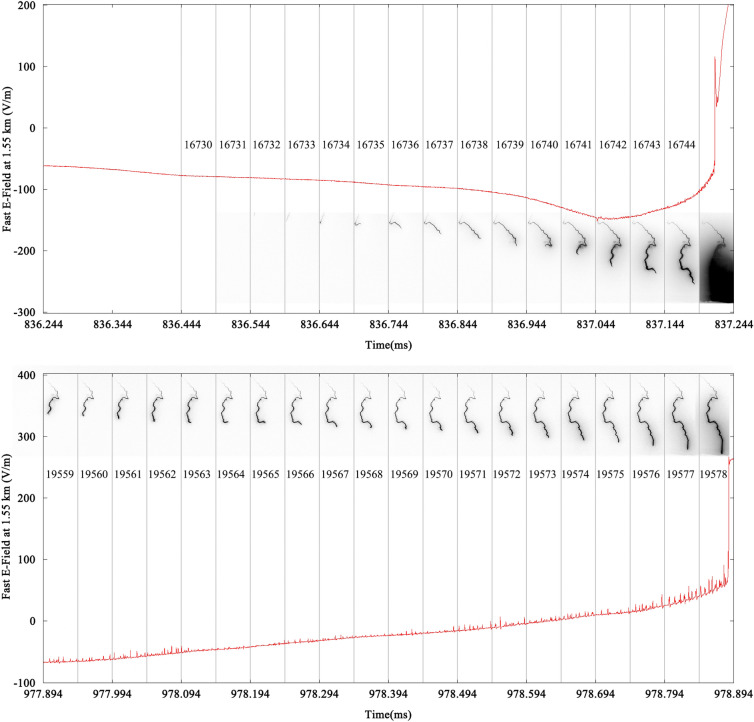
The electric field changes of DL13 (top) and DL14 (bottom) with corresponding high-speed frames. The waveform is separated by several grey vertical lines representing these 1 μs dead times to match high-speed frames.

Figure 5 illustrates the electric field changes of DL13 and DL14 with corresponding high-speed frames. The waveform in Fig. 5 is separated by several grey vertical lines representing these 1 μs dead times to match high-speed frames. The propagation of DL13 within FOV lasts less than 0.7 ms while that of DL14 lasts about 3.85 ms. The electric field waveform of DL13 is much smoother than that of DL14 with few pulses superimposed on it. There are only several minor pulses just before the beginning of RS13. DL14 has extensive distinct bipolar pulses and minor unipolar pulses superimposed on the long and slowly increasing electric field during the whole propagation process.

**Figure 6:**
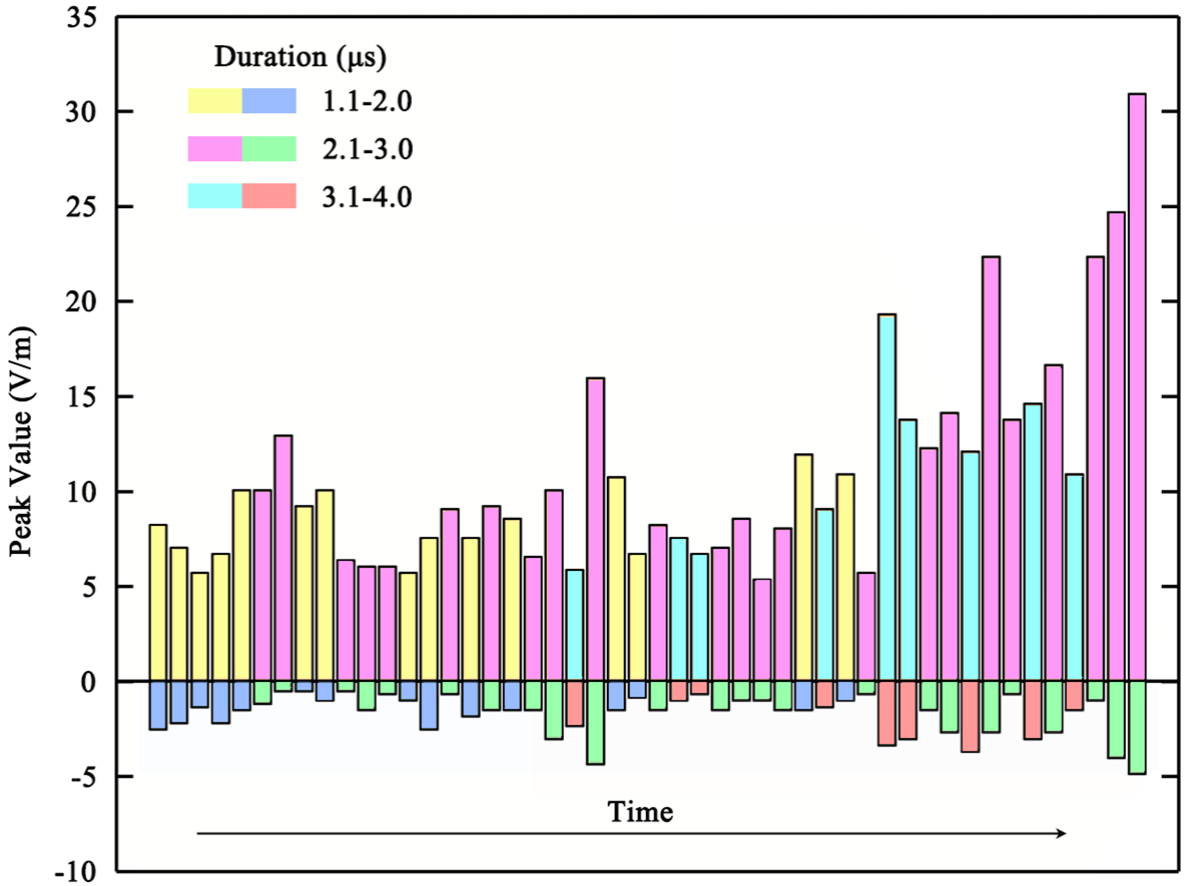
Features of 48 distinct bipolar pulses superimposed on the electric field at 1.55 km of DL14. The positive and negative peak values of these pulses are reflected by columns with colors representing their durations.

Figure 6 shows the features of 48 distinct bipolar pulses occurring during the development of DL14. The positive peak values of these bipolar pulses vary from 5.38 to 30.91 V/m with the mean value of 10.82 V/m. The negative peak values range from 0.50 to 4.87 V/m with the mean value of 1.79 V/m. The durations range from 1.1 to 3.6 μs with the mean value of 2.46 μs. Half of these 48 distinct bipolar pulses last 2.1 to 3.0 μs, and durations of these pulse tend to increase with time.

The pulses superimposed on the E-field of DL14 are different from those of DL4. Firstly, the pulses of DL14 consist of minor unipolar positive pulses and distinct bipolar pulses with higher positive peak value, while the pulses of DL4 are small unipolar positive pulses. Secondly, the pulses of DL14 are extensive during the whole propagation process, while the pulses of DL4 are scarce before the beginning of the bidirectional propagation process. Finally, there are over 48 distinct bipolar pulses having a positive peak value of over 5.38 V/m on the E-field of DL14, while those small positive pulses of DL4 have positive peak value of less than 4.36 V/m. Most of distinct bipolar pulses last over 2.0 μs, while all small positive pulses last less than 2.0 μs.

### Propagation speeds

As stated before, based on the polarity, propagation way and direction, there are five types of leaders in F1907021515: upward positive stepped leader (IUPL), upward positive dart leader (SUPL), downward negative dart leader (DL1 to DL3, DL5 to DL9, DL11 and DL13), bidirectional leader (DL4) and downward negative dart-stepped leader (DL10, DL12 and DL14). This provides a good chance to investigate various factors affecting the propagation speeds of these leaders.

**Figure 7:**
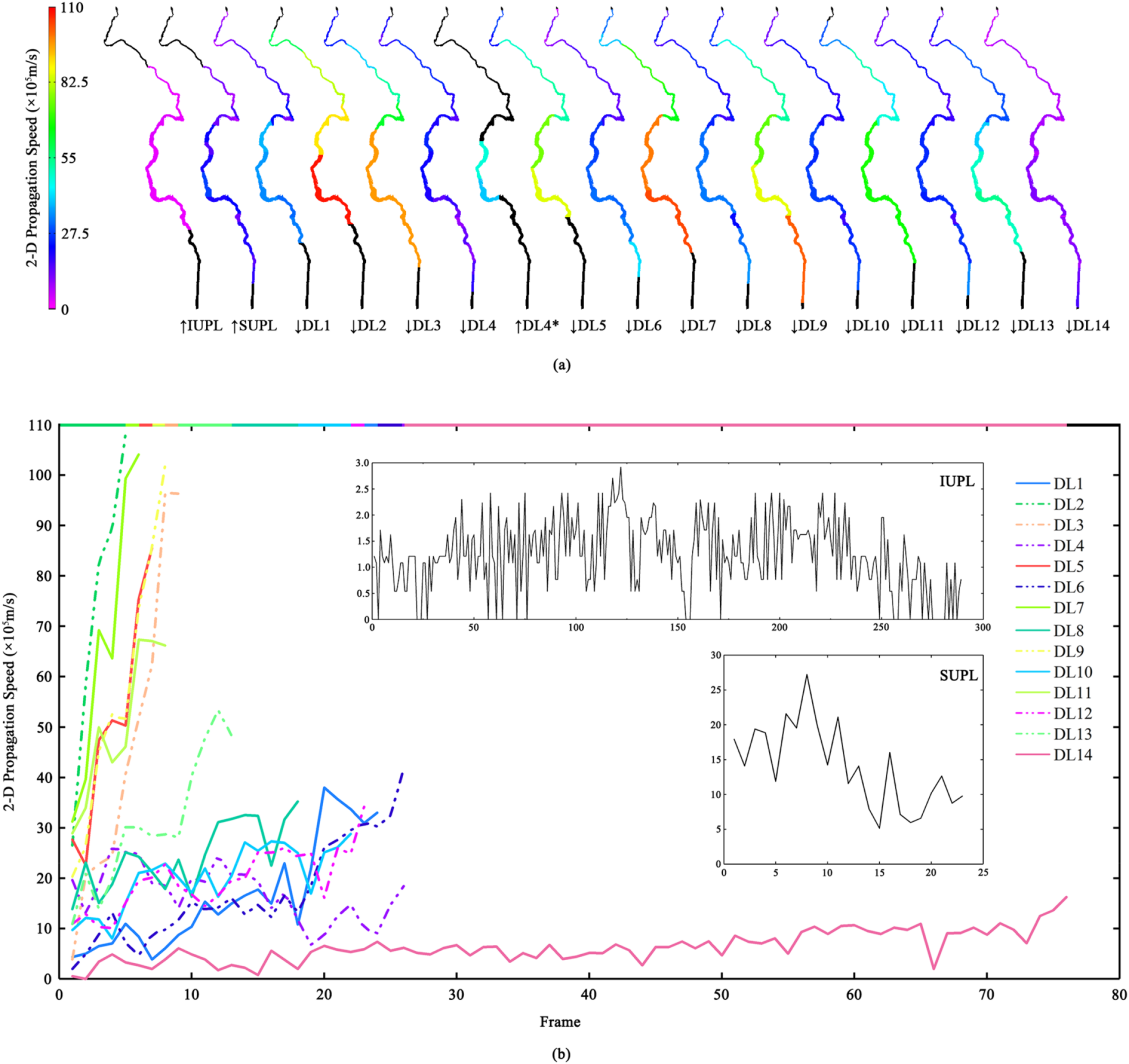
Propagation speeds variations of sixteen leaders in F1907021515. (**a**) The speed-position figure of sixteen leaders. The arrow preceding ID of each leader indicates the propagation direction. The upward propagation of bidirectional leader DL4 is also shown. (**b**) The propagation speeds variations of sixteen leaders. Only the downward propagation of bidirectional leader DL4 is shown. The top border is colored to show the duration of each leader.

Figure 7 shows the propagation speeds of the sixteen leaders. Figure 7a colors these channels from pink to red to reflect the propagation speeds variation of each leader. Figure 7b illustrates these propagation speeds variation in a traditional way. The two upward leaders (IUPL and SUPL) are different from other downward leaders in that the speeds of them fluctuate intensively without a clear trend. And over time, the speeds of these two upward leaders seem to decrease. The reason why upward leaders seem to slow down while downward leaders accelerate with time may be attributed to the inherent defect of the 2-D observation. Specifically, the upper portion of the channel is probably farther from the observation station than the bottom. During the propagation of SUPL, the electric field turns to decrease before SUPL leave FOV, which indicates that SUPL is probably propagating away from the observation station as the height increases. When we calculate the 2-D propagation speeds, the whole channel is supposed to be 1.55 km away from the observation station. Therefore, the final speeds of upward leaders or the initial speeds of downward leaders are probably underestimated, which results in the observed deceleration of upward leaders and acceleration of downward leaders.

Compared with IUPL, SUPL has obviously higher speeds. When IUPL propagates upward, the head of it pauses at some certain positions leading speeds here to be 0. SUPL propagate continuously without any noticeable pauses. IUPL propagates at speeds lower than 2.34 × 10^5^ m/s, while SUPL propagates at speeds higher than 5.14 × 10^5^ m/s and an average speed on the order of 10^6^ m/s. Propagating in the same direction, the upward end of BL (DL4) has two speed values calculated to be 39.60 and 46.11 × 10^5^ m/s, higher than those of both IUPL and SUPL. The higher speeds of the upward end of BL probably benefit from the better channel condition left by the initial decaying downward propagation. Two BLs starting almost immediately below the termination points of a decaying dart or dart-stepped leader were reported by Qie et al.^16^. The two BLs has an upward end propagating with an average speed of 1.3 × 10^6^ and 2.2 × 10^6^ m/s, which are slower than the upward end of BL here. The BL here is transformed from a decaying but not terminated unidirectional leader while those two BLs reported before started after the unidirectional leader died out. The much shorter interval may explain why the upward end here has a higher speed.

These fourteen downward leaders can be classified into two kinds based on their speeds variations: acceleration and fluctuation, as shown in Table 1. The difference between these two kinds of leaders is clearly shown in both Fig. 6a,b. The acceleration leaders have shorter durations and higher speeds with a clear and sharp acceleration. The colors of their channel change from pink to red from top to bottom. The fluctuation leaders have longer durations and lower speeds with a more zigzag speeds variation. The colors of their channel range from pink to blue. The quantitative difference between these two kinds of leaders is whether a leader has a speed boost over 2 × 10^6^ m/s and doesn’t have a speed lost over 1 × 10^6^ m/s.

The acceleration leaders here are all dart leaders, not vice versa. DL1, DL6 and DL8 are all dart leaders having a smooth electric field waveform with few pulses and an average speed on the order of 10^6^ m/s but belonging to the fluctuation leaders. The three dart-stepped leaders (DL10, DL12 and DL14) and the bidirectional leader DL4 are all fluctuation leaders. The occurrence of fluctuation leaders seems to have no clear relationship with the interstroke interval, i.e. the fluctuation leaders do not always occur after a longer interstroke interval. A clear relationship can be found between these two kinds of leaders is that the leader occurring after a fluctuation leader is always an acceleration leader. That’s to say, acceleration leaders can occur in succession while fluctuation leaders cannot.

## Discussion

Typical negative classical triggered lightning flashes or upward lightning flashes initiated from high structures involve two or three types of lightning leaders, i.e. upward positive stepped leaders and downward negative dart leaders or downward negative dart-stepped leaders. A forth type of leader, upward positive dart leader, is observed for the first time by Li et al.^15^. The cases of triggered lightning flashes involve a fifth type of leader, BL, in the residual channel are only reported by Qie et al.^16^. In this paper, we have found a negative triggered lightning flash containing all these five types of lightning leaders.

Li et al.^15^ found the 3-D speeds of the upward positive dart leader (referred to as UPL* below) had a range from 3.72 × 10^6^ to 14.48 × 10^6^ m/s, with the mean value of 6.5 × 10^6^ m/s. When it propagated between 0 to 2 km above the ground, UPL* had three decreasing 2-D speed values calculated, all of which are between 3 × 10^6^ and 5 × 10^6^ m/s. SUPL here is recorded to propagate between 0 to 1.6 km above the ground and have fluctuating 2-D speed values between 5.14 × 10^5^ and 2.72 × 10^6^ m/s, which is much slower than UPL*. The UPL* occurred 5.8 ms after the preceding RS, while the SUPL occurs about 30 ms after the last ICCP. The slower propagation speeds of SUPL are probably caused by the much longer interstroke interval, which leads the channel condition of the residual channel to be worse.

Li et al.^15^ proposed that the better channel condition, the larger enough background electric field and the residual negative charges after RS4 are the key factors in triggering UPL* without any preceding in-cloud discharge activities. The residual negative charges left in the residual channel in that case was believed to be caused by the cutoff of the preceding RS4 current, i.e. the downward dart leader deposited more negative charge than the RS4 current actually neutralized later. Similar to UPL*, SUPL here also occurs after a decrease of the electric field.

The inference proposed by Li et al.^15^ well explained why UPL* occurred. However, why are upward stepped leaders initiated in virgin air more often observed in upward lightning flashes while upward dart leaders initiated in the residual channel with better channel conditions are rare? After being initiated, a RS is often observed to retrace and illuminate the longer channel than the preceding downward leader and sometimes illuminate the channel of decayed branches^18^. The upward mini-RS observed in altitude-triggered lightning flashes will also enhance the upward propagation of the IUPL and make it easier to make branches^19^. These indicate that RSs usually neutralize more negative charge than preceding downward leaders deposited on the channel, if the RS current cutoff doesn’t happen. With the RS current gradually decays, more and more positive charge is deposited on the residual channel, which prevents the following potential initiation of upward positive dart leaders. In the case of UPL* and SUPL here, the preceding decreasing electric field indicates that there exist some discharge processes enhancing the negative background electric field and offsetting the inhibitory effect of those positive charge. Upward connecting leaders, which are induced by the approaching downward leaders, are difference from the upward dart leaders propagating continuously to start a CC discussed here.

Qie et al.^16^ first reported two BLs (referred to as BL1* and BL2* below) in the residual channel of triggered lightning flashes. BL1* started about 160 μs after the termination of the preceding dart leader at the height of 452 m above the ground. BL2* started about 300 μs after the termination of the preceding dart leader at the height of 339 m above the ground. In this paper, DL4 has a similar decaying propagation with the leaders before the initiation of BL1* and BL2*. However, DL4 never dies out and has a luminous enough channel longer than 250 m before the bidirectional propagation process. DL4 directly turns to be a bidirectional leader from a unidirectional leader when the head of it descends to the top of exploded triggering wire channel, i.e. the height of about 280 m above the ground. The electric field changes of DL4 also has the two features, slow change and small positive pulses, proposed by Qie et al.^16^ for the fast electric changes of bidirectional leader progression.

During the DL4 process, the downward leader kept developing downward, indicating that the channel has a large enough conductance for the development of the leaders. The main reason why the upper channel of the leader decayed when the leader was propagating downward was the lack of the charge transformed from the cloud sources. The downward leader was getting closer to the ground and the leader developed to the channel position of the previous vaporized rocket wire with a higher conductance, making the negative leader with insufficient charge transmission stopped decaying and accelerated. The net charge of the leader channel separated from the cloud was approximately unchanged. The emergence of negative downward leader led to the emergence of upward positive leader, which made the generation of the bidirectional leader. The residual negative charge in the upper decayed channel also promoted the generation of the upward positive leader.

The propagation of DL4 proves that a bidirectional leader can be transformed from a decaying unidirectional leader, and bidirectional leaders occurring in this way also fit the fast electric changes mode proposed for bidirectional leaders starting below the termination point of an attempted leader. Qie et al.^16^ suggested that those small positive pulses are caused by the potential stepwise downward propagation of BL1* and BL2*. However, as analyzed in Sect. 3.4, these small positive pulses are quite different from pulses caused by stepwise propagation of dart-stepped leaders. In this paper, the bidirectional leader DL4 and the dart-stepped leader DL14 propagate along the same path and are recorded by the same equipment, and hence the differences between pulses of them prove that these small positive pulses are not results of the potential stepwise downward propagation of BLs.

Three dart-stepped leaders and the bidirectional leader here are all classified as fluctuation leaders which have short durations, relatively low propagation speeds without a clear and sharp acceleration. Some dart leaders here also belong to fluctuation leaders, which means they are not different from dart-stepped leaders in terms of progressing characteristics. Therefore, the existence of abundant pulses superimposed on the electric field changes or the observation frame rate high enough to record the step formation are vital tools to judge whether a specific leader is a dart-stepped leader or a dart leader.

It can be found in Fig. 1 that all RSs induced by fluctuation leaders have higher positive peak values than RSs induced by acceleration leaders in electric field changes recorded at 130 m (positive peak values of RSs at 1.55 km are all saturated), as revealed by the black horizontal dotted line. This indicates that RSs induced by fluctuation leaders have higher speeds. The stepped leaders are generally considered to have a larger electric potential and bring a more pronounced attachment process than the subsequent dart leaders. The fluctuation leaders here share a similar fluctuating speeds variation with stepped leaders and result in RSs with higher speeds. The higher speeds of RSs induced by fluctuation leaders may final result in more positive charge left in the residual channel, making it reasonable that the leader occurring after a RS induced by a fluctuation leader is always an acceleration leader. That’s to say, the positive charge left in the residual channel can not only inhibit the initiation of an upward positive leader but also boost the propagation of a downward negative leader.

**Figure 8:**
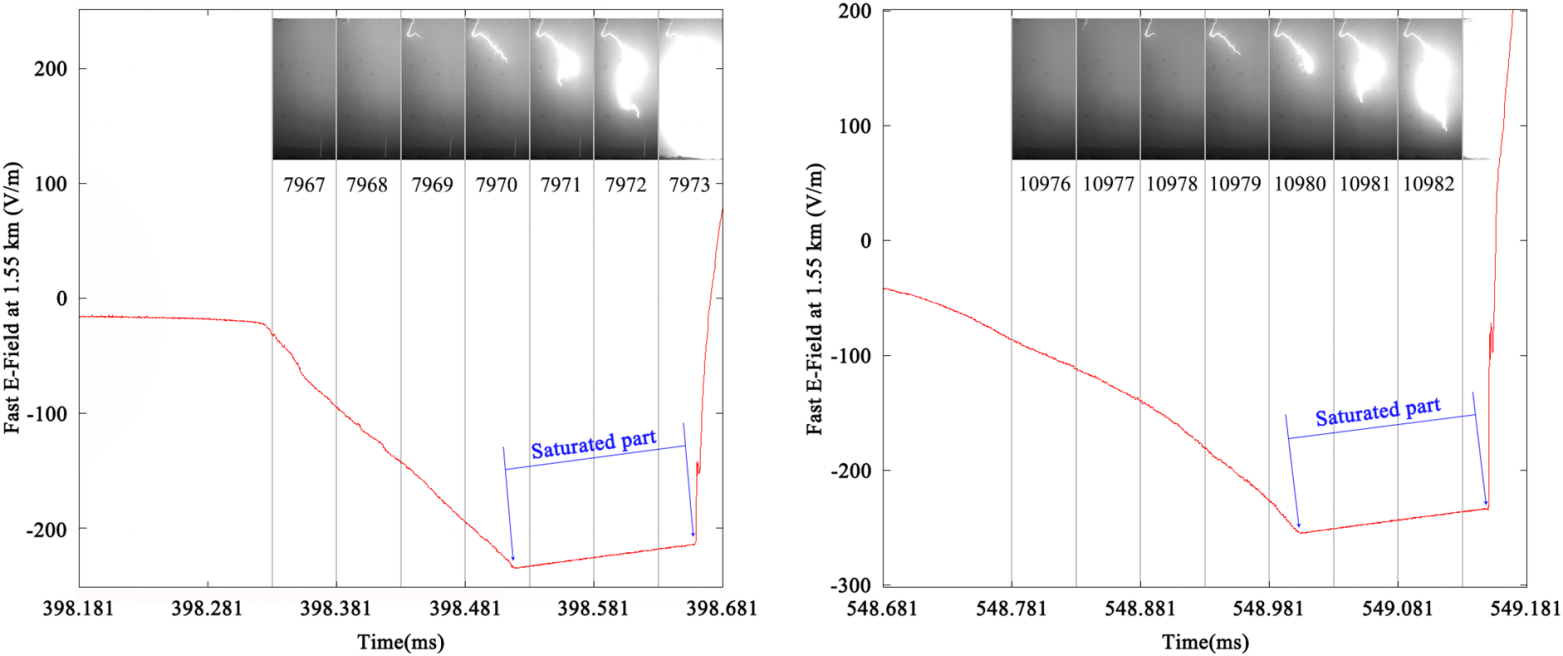
Two examples of acceleration leaders whose propagation result in saturation of both high-speed frames and electric field recorded at 1.55 km. The left one is DL2 and the right one is DL7. The electric field at 1.55 km and brightness of high-speed frames get saturated simultaneously. The exposure time of each high-speed frame is 49 μs.

More intuitive phenomenon supporting the existence of positive charge in the residual channel is that all acceleration leaders here become overexposed on a large scale in the recorded high-speed frames using an exposure time of 49 μs before attachment processes. Figure 8 shows two of these acceleration leaders whose propagations result in saturation of both high-speed frames and electric field recorded at 1.55 km. The high-speed frames usually get overexposed on a large scale only when the attachment processes begin or RSs are initiated during the development of a negative triggered lightning flash. The frames getting overexposed earlier suggest that an intense enough discharge process has already happened before these acceleration leaders induce RSs. This intense enough discharge process is probably caused by the existence of positive charge in the residual channel, and the neutralization of positive and negative charge leads to the lower positive peak values of RSs induced by acceleration leaders.

## Methods

### Guangzhou triggered lightning flashes observation experiment

The negative triggered lightning F1907021515 was triggered at 07:15:04 UTC on 2 July 2019 at the Guangzhou Field Experiment Site for Lightning Research and Testing in Conghua, Guangdong Province, China. The observations were performed by Engineering Research Center of Lightning Protection & Grounding Technology, Ministry of Education, China. More detailed information about experiments carried out here can be found in Cai et al.^20^. F1907021515 was triggered using the classical technique and recorded by a high-speed camera and fast electric field antennas at different distances.

### High-speed camera

The high-speed camera used here is a Phantom v2512 high-speed camera operating at a frame rate of 20 kfps, with an exposure time of 49 μs per frame. The size of each pixel was 28 μm × 28 μm, and the image resolution was 640 × 608 pixels (horizontal × vertical). The high-speed camera was coupled with a Nikon 16 mm lens at f/2.8 and located on the roof of a five-story building positioned 1.55 km south to the launch tower. At this distance, the spatial resolution was about 2.71 m per pixel, and the field of view (FOV) was about 1734 m horizontally and 1648 m vertically.

### Electric field signal measurement

The electric field signal at 130 m was measured by the fast antenna set to be about 3.6 m above ground and recorded by a multi-channel high-speed digital oscilloscope (DL850E) with a sampling rate of 50 MHz and a recording length of 2 s. The electric field signal at 1.55 km was measured by the fast antenna installed beside the high-speed camera and recorded at a sampling rate of 5 MHz. Wide-band electric field measuring system (with time constant of 1 ms and a 3 dB bandwidth from 160 Hz to 500 kHz) are employed to measure the lightning electric field changes at these two distances.
